# Mantle Cell Hyperplasia of Peripheral Lymph Nodes as Initial Manifestation of Sickle Cell Disease

**DOI:** 10.1155/2016/8507317

**Published:** 2016-10-30

**Authors:** Ahmad Monabbati, Sadat Noori, Akbar Safaei, Mani Ramzi, Seyedsajjad Eghbali, Ali Adib

**Affiliations:** ^1^Department of Pathology, Shiraz University of Medical Sciences, Shiraz, Iran; ^2^Department of Hematology-Oncology and Stem Cell Transplantation, Shiraz University of Medical Sciences, Shiraz, Iran; ^3^Department of Pathology, Bushehr University of Medical Sciences, Bushehr, Iran; ^4^Student Research Committee, Shiraz University of Medical Sciences, Shiraz, Iran

## Abstract

Sickle cell disease (SCD) is a well known hemoglobinopathy with usual manifestations including anemia, hyperbilirubinemia, and vasoocclusive complications. Despite presence of mild splenomegaly in early phase of the disease, lymphadenopathy is not an often finding of SCD. We introduce an undiagnosed case of SCD who presented in third decade of his life with multiple cervical lymphadenopathies and mild splenomegaly persistent for about five years. Histopathologic examination of the resected lymph nodes showed expansion of the mantle cell layers of secondary follicles as well as several monomorphic mantle cell nodules. To rule out possibility of a malignant process involving lymph nodes, an immunohistochemical panel was ordered which was in favor of benign mantle cell hyperplasia. Immunoglobulin gene rearrangement study showed no clonal bands and confirmed benign nature of the process. Respecting mild abnormalities on Complete Blood Count, peripheral blood smear was reviewed revealing some typical sickle red blood cells as well as rare nucleated red blood cells. Solubility test for hemoglobin (HB) S was positive. Hemoglobin electrophoresis confirmed diagnosis of homozygous HbS disease.

## 1. Introduction

Sickle cell disease (SCD) is an important hereditary hemoglobinopathy which affects more than 300000 annual births worldwide mostly in low- and middle- income countries [1].

Its usual clinical manifestations include anemia, hyperbilirubinemia, and vasoocclusive complications [2]. The patients experience some episodes of painful crisis resulting from acute vasoocclusive pain due to entrapment of erythrocytes and leucocytes in the microcirculation [3].

While peripheral blood smear shows anemia associated with presence of some sickle red blood cells (RBC), target cells, and a few nucleated RBCs, bone marrow is hyperplastic because of compensatory hyperplasia of erythroid precursors. Mild splenomegaly is seen during childhood, in early phase of disease. Thereafter, progressive shrinkage of the spleen due to continuous scarring occurs by adolescence or early adulthood, a process called autosplenectomy [4]. Premature mortality occurs due to the complications of this disease, especially cardiopulmonary organ dysfunction and chronic kidney injury [5].

This article presents a 22-year-old boy newly diagnosed with SCD who had cervical lymphadenopathy as an atypical sign.

## 2. Case Presentation

A 22-year-old male presented with persistent multiple cervical lymphadenopathy that had gradually developed in size, mild splenomegaly, and several small lymph nodes in perisplenic area for about five years. He had past history of episodes of painful crisis in upper and lower limb bones since childhood which resolved by analgesic but he never sought medical treatment.

Due to persistent lymphadenopathy, splenomegaly, and clinical suspicion of lymphoma, the largest cervical lymph node measuring 2.5 × 1.5 × 1 cm was excised with initial pathologic report of reactive follicular hyperplasia. It was referred to our center for consultation. Lymph node showed well preserved architecture containing secondary follicles with pale staining germinal centers (GCs) surrounded by dark staining layer of mantle cells. Mantle cell proliferation resulting in broad and expansive mantle zone layer was evident in some of the follicles.

**Figure 1:**
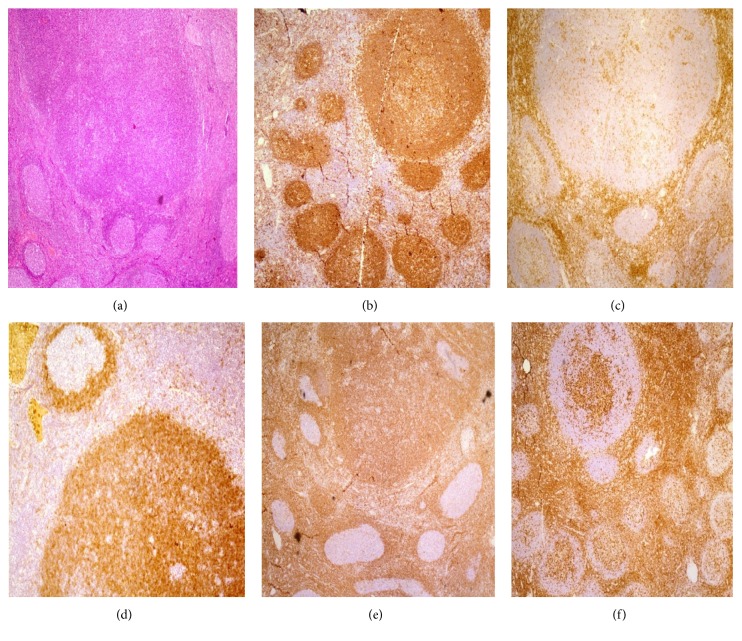
Sections from an enlarged cervical lymph node. (a) Hematoxylin and Eosin staining. (b)–(f) Immunohistochemical staining for CD20 (b), CD5 (c), IgD (d), Bcl-2 (e), and CD43 (f).

Several monomorphic nodules composed of small sized lymphocytes without germinal center were also noted mainly in cortical region of the lymph node. Interfollicular region was unremarkable (Figure 1(a)). No invagination of mantle layer inside germinal centers and transformation of the layer were identified.

On closer inspection, the nodules were composed mainly of small sized lymphocytes with round to very slightly indented nuclei and scant amount of cytoplasm.

Several congested capillaries and venules containing deformed RBCs with a few typical sickle cells were also noted in the sections after reviewing.

The main differential diagnoses were mantle zone hyperplasia, early stage of mantle cell hyperplasia, or other low grade lymphomas. Therefore, immune histochemistry (IHC) was done to determine true nature of the process. Briefly after quenching of endogenous peroxidases and heat induced antigen retrieval, sections were incubated with mouse monoclonal antibodies against CD20, Ki67, Bcl-2, and CD43 (1/300, predilute, and 1/80, resp.; Dako Corp, Carpinteria, CA, USA), mouse monoclonal antibodies against CD3, CD5, and CD23 (1/100, 1/5, and predilute, resp.; Novocastra Lab. Ltd. UK), rabbit polyclonal antibodies against Immunoglobin (Ig) D, IgM, IgG, and Kappa and Lambda light chains (1/3.5, 1/5000, 1/14000, 1/10000, and 1/10000, resp.; Dako Corp, Carpinteria, CA, USA), and rabbit monoclonal antibody against Cyclin D1 (1/80; Dako Corp, Carpinteria, CA, USA). Bound primary antibodies were reacted with secondary antibodies conjugated with horse radish peroxidase. Visualization of the complexes was accomplished with DAB as chromogen. Sections were also lightly stained with Mayer's hematoxylin. Hyperplastic follicles, expanded mantle zone layers, and the monomorphic nodules were strongly positive for CD20, CD19, and Pax-5. Immunostaining with CD3 highlighted interfollicular T-cell areas which were unremarkable. Ki67 staining showed high proliferation activity confined to the GCs whereas the areas outside the GCs and the monomorphic B-cell nodules had low proliferation activity (<5%). Bcl-2 was not detected in GCs. Mantle cell layer of the follicles and the monomorphic B-cell nodules were diffusely positive for IgD and Bcl-2 whereas positivity for CD23 was weak and scattered. Reaction for IgM was also weakly positive (Figures 1(d) and 1(e)). Immunostaining for CD5, CD43, and Cyclin D1 was negative in both mantle cell layer of the hyperplastic follicles and the B-cell nodules (Figures 1(c) and 1(f)). Immunohistochemical tests for Kappa and Lambda light chain restriction were inconclusive.

To evaluate the clonality of the lymphoid population, immunoglobulin heavy chain gene rearrangement study was performed on DNA, extracted from 10 five-micron-thick sections of FFPE tissue, using proteinase K incubation method followed by phenol-chloroform extraction protocol. Conventional PCR for Ig gene rearrangement study was performed using BIOMED-2 concerted action primers from InVivoScribe Technologies (San Diego, CA) based on manufacturer's instructions. DNA integrity was controlled using three primer sets for amplification of beta-globin gene with PCR products of 110 bp to 400 bp.

PCR products were detected and analysed using polyacrylamide gel 6% and heteroduplex assay. In the presence of beta-globin gene amplification, conventional PCR for Ig gene rearrangement study did not yield any clonal bands.

Even though lymphadenopathy in this patient was a long lasting sign, a comprehensive investigation for possible infectious etiologies was done and no cause for this lymphadenopathy was found (Table 1). Also, no morphological change indicative for specific infectious etiologies was found in lymph node tissue sections.

Searching for probable causes, all data were reviewed which showed anemia with mild leukocytosis in Complete Blood Count (CBC).  Table 2 shows the results of hematologic studies. Also mild splenomegaly appeared in abdominal sonography.

Regarding leukocytosis, peripheral blood smear was prepared which revealed presence of some typical sickle RBCs, a few target cells, and rare nucleated RBCs. No atypical lymphoma or leukemia cell was identified. Solubility test for hemoglobin (Hb) S was strongly positive and Hb electrophoresis showed homozygous HbS disease with >80% HbS, >10% HbF, and <5% Hb A1.

## 3. Discussion

Our report describes a young male who presented with persistent multiple cervical lymphadenopathy and mild splenomegaly for about 5 years. Histomorphologic review of the resected lymph node showed well defined secondary follicles as well as several monomorphic nodules composed of monotonous population of small lymphocytes mainly with regular round nuclei, inconspicuous nucleoli, and scant cytoplasm.

Expanded mantle zone of secondary follicles and the nodular proliferations showed positive reaction for CD20, IgD, Bcl-2, IgM (weak), and CD23 (scattered) along with negative immunostaining for CD3, CD5, CD43, and Cyclin D1. This pattern of IHC profile is characteristic of benign mantle cells which are responsible for proliferation and expansion producing broad mantle cell layers and mantle cell nodules in this case. Reactive follicular hyperplasia of any other cause, reactive follicles with transformation of germinal centers, marginal zone hyperplasia, and early stage of mantle cell lymphoma are the main differential diagnoses of such finding.

It is acceptable that mild lymphadenopathy be seen in SCD due to abnormal shape and sickling of RBCs occurring in various hypoxic conditions. Patients with autosplenectomy are susceptible to infectious diseases that can cause lymphadenopathy and lymph node enlargement [6]. Also, it is found that the children with SCD have increased lymphoid tissue in the head and neck region, manifested by prominence of the adenoids and palatine tonsils, in proportion to general population [7]. However, initial presentation of SCD in third decade of life with persistent lymphadenopathy due to mantle cell hyperplasia has not been reported yet. Mantle cell hyperplasia is a rare reactive change seen in lymph node. It has been only rarely reported so far and the main causes of it are not well known [8].

Lymphadenopathy in this case is most likely due to the disease process itself. By considering persistence of the problem for about 5 years and absence of pertinent clinical and paraclinical data denoting specific etiologic factors, lymphadenopathy caused by mantle cell hyperplasia can be considered a finding directly related to SCD and the first presentation of the disease in this case. Besides these, mantel cell hyperplasia is not a usual well known response to microbial agents. We proposed that sequestration of node sinuses by sickle cells without vascular occlusion and secondary infarction may elicit an immunologic response leading to this peculiar lymphoid hyperplasia. Also, chronic nonspecific immune stimulation and adenitis may be another explanation for persistent lymph node enlargement leading to initial diagnosis of SCD.

## Figures and Tables

**Table 1:** Viral studies and serum immunoglobulin level on admission time.

Laboratory parameters	On admission
VCA-IgG for EBV	Positive
VCA-IgM for EBV	Negative
Monospot test for EBV	Negative
HBS antigen	Negative
HBV IgG	Negative
HCV IgG	Negative
HIV IgG	Negative
CMV IgM	0.7 IU
CMV IgG	1.5 IU
IgG serum level (gr/dL)	2
IgM serum level (gr/dL)	0.25
IgA serum level (gr/dL)	0.30

**Table 2:** Results of hematologic studies.

Laboratory parameters	On admission
Hemoglobin (g/dL)	11.2
Red blood cell (count/mm^3^)	4220000
Mean corpuscular volume (fL)	84.6
White blood cell (count/mm^3^)	13100
Neutrophil (%)	61
Lymphocyte (%)	25
Mixed (%)	14
Platelet (count/mm^3^)	259000
Prothrombin time (sec)	13
International normalized ratio	1
Partial thromboplastin time (sec)	35
Erythrocyte sedimentation rate (mm/hr)	5
Blood urea nitrogen (mg%)	23
Creatinine (mg%)	1.2
Total bilirubin (mg%)	3.2
Direct bilirubin (mg%)	0.3
C-reactive protein (mg/dL)	Negative
